# Ultrapure dialysis water obtained with additional ultrafilter may reduce inflammation in patients on hemodialysis

**DOI:** 10.1007/s40620-017-0422-x

**Published:** 2017-08-23

**Authors:** Biagio Di Iorio, Lucia Di Micco, Dario Bruzzese, Luca Nardone, Luigi Russo, Pietro Formisano, Vittoria D’Esposito, Domenico Russo

**Affiliations:** 1 Department of Nephrology, “A. Landolfi” Hospital (Solofra, Avellino), Solofra, Italy; 2 Department of Statistics, University of Naples “FEDERICO II”, Naples, Italy; 3 Department of Public Health, University of Naples “FEDERICO II”, Naples, Italy; 4 Department of Genetics, University of Naples “FEDERICO II”, Naples, Italy

**Keywords:** Ultrafilter, Dialysis water, Ultrapure water, Dialysis patients, Inflammation, Cytokines, Erythropoietin stimulating agents

## Abstract

**Background:**

Patients on standard dialysis, in particular those on high-flux and high-efficiency dialysis, are exposed to hundreds of liters of dialysis-water per week. The quality of dialysis-water is a factor responsible for inflammation in dialysis patients. Inflammation is a potent trigger of atherosclerosis and a pathogenetic factor in anemia, increasing mortality and morbidity in dialysis patients. Current systems for water treatment do not completely eliminate bacteria and endotoxins. This prospective study tested whether improved dialysis-water purity by an additional ultrafilter can reduce inflammation and ameliorate hemoglobin levels, with a consequent reduction in erythropoietin-stimulating agents (ESA).

**Methods:**

An ultrafilter, composed of two serially positioned devices with polysulfone membranes of 2.0 and 1.0 m^2^, respectively, was positioned within the fluid pathway before the dialysis machine. Prevalent dialysis patients were assigned either to continue dialysis with conventional dialysis-water (control phase) or to initiate dialysis sessions with improved dialysis-water purity (study phase). After 6 months, patients were crossed over. Total study duration was 1 year. Routine chemistry, bacterial count, endotoxin levels in dialysis-water as well as blood levels of pro- and anti-inflammatory cytokines, human serum amyloid A, C-reactive protein and fraction 5 of complement were measured.

**Results:**

Thirty-two patients completed the study. Mean bacterial count was lower and endotoxin levels were absent in dialysis-water obtained with the ultrafilter. At the end of the study-phase, C-reactive protein and pro-inflammatory cytokines decreased while anti-inflammatory ones increased. Hemoglobin levels were improved with lower ESA doses.

**Conclusions:**

An additional ultrafilter improved dialysis-water purity, reduced levels of inflammation markers, ameliorated hemoglobin concentration with reduced ESA doses. These results remain speculative but they may generate studies to assess whether improved dialysis-water quality with an ultrafilter can reduce inflammation and improve survival of dialysis patients.

**Electronic supplementary material:**

The online version of this article (doi:10.1007/s40620-017-0422-x) contains supplementary material, which is available to authorized users.

## Introduction

Inflammation plays a critical role in mortality and morbidity of dialysis patients, being a potent trigger of atherosclerosis and a pathogenetic factor in anemia [1–5]. There are several factors linked to uremia that can cause inflammation; some can be regarded as modifiable risk factors. This is the case of quality of dialysis-water. Current systems for water treatment do not completely remove bacteria and endotoxins [6–8]. Therefore, the quality of dialysis-water may be a significant factor responsible for inflammation. Indeed, patients on conventional dialysis, in particular those on high-flux and high-efficiency dialysis, are exposed to hundreds of liters of dialysis-water per week. Therefore, every effort should be made by clinicians to improve the purity of dialysis-water in order to minimize the negative effects of inflammation.

This study aimed to test in patients on dialysis whether an additional ultrafilter can decrease blood levels of markers of inflammation, increase hemoglobin levels (Hb) and reduce doses of erythropoietin-stimulating agents (ESA).

## Methods

This was a prospective, crossover study in prevalent dialysis patients. Patients signed written informed consent. The local ethic committee approved the study. Exclusion criteria were: inflammatory diseases; therapy with anti-inflammatory drugs or steroids; signs of malnutrition such as abnormal serum levels of albumin, cholesterol or triglycerides and change in dialysis prescription or ESA therapy within the 3 months preceding the study.

**Fig. 1:**
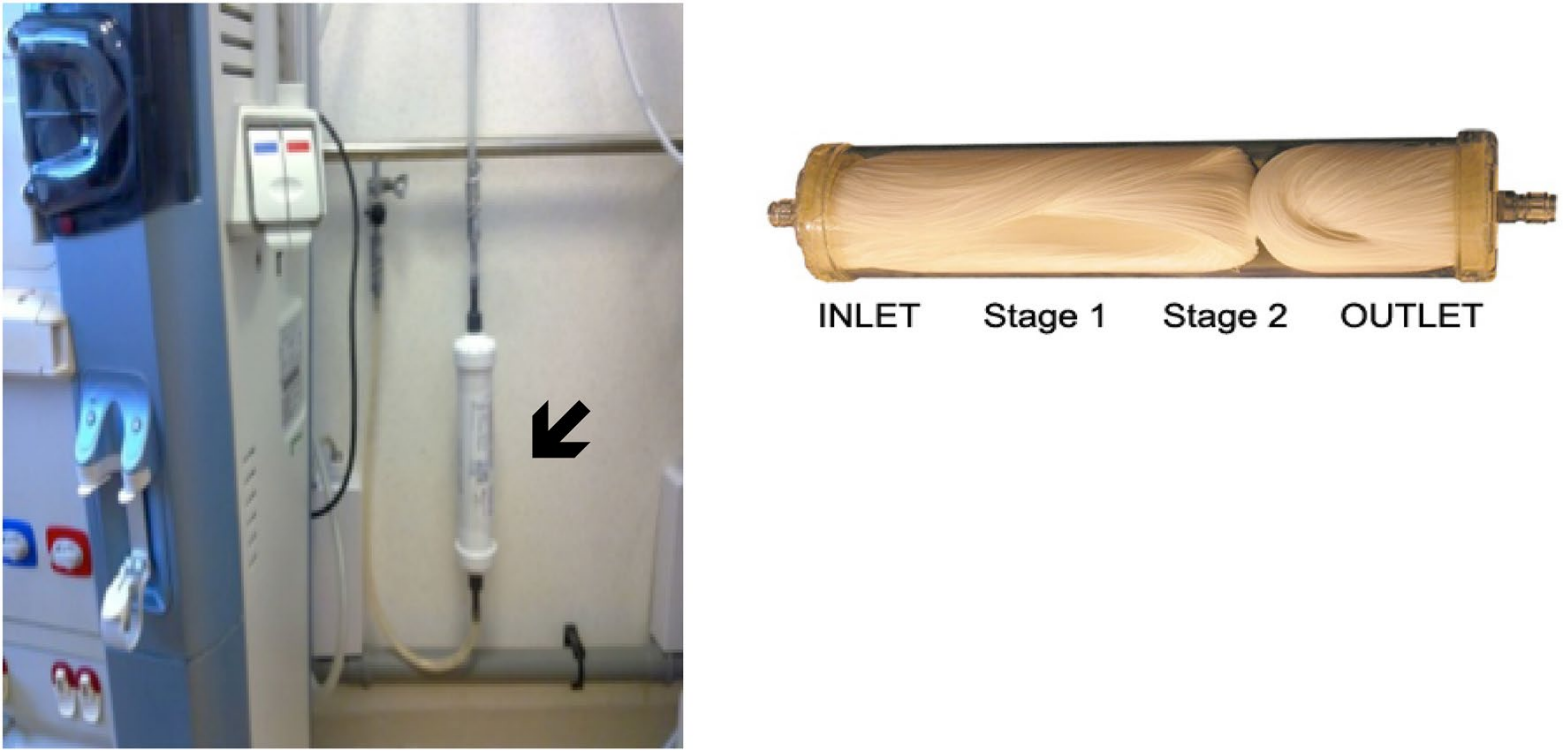
Composition and position of ultrafilter. The ultrafilter was composed of two serially positioned devices with polysulfone membranes [MediSulfone, Medica, Medolla (MO), Italy] of 2.0 and 1.0 m^2^, respectively. Characteristics of the ultrafilter were: bacteria retention capacity >1010 CFU/ml (*Brevundimonas diminuta*), viruses retention capacity >108 (PhiX 174), endotoxin retention capacity >105 EU/ml

To improve the dialysis-water quality, an ultrafilter (Estorclean PLUS) was positioned within the fluid pathway before the dialysis machine. The ultrafilter was composed of two serially positioned devices with polysulfone membranes [MediSulfone, Medica, Medolla (MO), Italy] of 2.0 and 1.0 m^2^, respectively (Fig. 1). Characteristics of the ultrafilter were: bacteria retention capacity >1010 CFU/ml (*Brevundimonas diminuta*), viruses retention capacity >108 (PhiX-174), endotoxin retention capacity >105 EU/ml. The ultrafilter remained *in situ* for the duration of the study.

Each patient was assigned either to dialysis sessions with conventional dialysis-water (control phase) or to dialysis sessions with improved dialysis-water quality (study phase). After a 6-month observation period, each patient was switched to dialysis sessions with the other dialysis-water. The total study duration was 1 year. Routine chemistry, pro- and anti-inflammatory cytokines, tumor necrosis factor (TNF)-α, serum A-amyloid (SAA), and complement fraction 5 (C5a) were measured at the beginning and end of each study phase; blood samples were collected at the mid-week dialysis session. Mean duration of the dialysis sessions was 4 h, 3 times a week. The dialysis membranes were: polyphenylene (n = 14 patients), polycarbonate (n = 6), polymethylmethacrylate (n = 6), and polyamide (n = 6). Changes in dialysis membrane were not permitted during the study.

Dialysis-water was obtained by reverse osmosis. Water quality was checked bi-monthly by an independent laboratory (Department of Public Health, University of Naples “Federico II”). Total microbial count was measured, and the limulus amebocyte lysate test (LAL-test; Pyrotell^®^ Multitest Vial; Cape Cod Inc., Falmouth, MA, USA) was performed on samples collected from water entering into the dialysis machine. Three determinations were performed during both the control and study phase. A combination of chemical and heat disinfection of the dialysis machine was performed at the end of each patient treatment. A special disinfection cycle (lasting 45 min) was performed twice a month in each machine with heat and chemical disinfection.

For cytokines and growth factors (Bio-Rad, Hercules, CA, USA) serum samples were analyzed in duplicate and diluted 4×; for SAA and C5a (HyCult Biotech, Uden, Netherlands) samples were analyzed in duplicate and diluted 40× and 100×, respectively. Dilutions were performed with the specific dilution buffer provided by the manufacturers.

## Statistical analysis

Numerical variables are summarized using median; categorical variables are described using absolute frequencies and percentages. In order to properly account for the crossover study-design and skewed distribution of variables, the non-parametric approach was used. In particular, the presence of a difference in carry-over effect was first evaluated using the Wilcoxon rank-sum test on the subject totals (summing up the two periods) while the significance of treatment effect was assessed with the Wilcoxon rank-sum test on the subjects’ period differences. Corresponding point estimates and confidence intervals (CI) were computed using the Hodges and Lehman estimator. Finally, non parametric analysis of the cross-over difference allowed us to assess the presence of a period effect. The decision to use a non parametric approach for the analysis of the cross-over design was related to the markedly skewed distribution of the outcome variables. However, the inferential properties of non parametric tests remain the same as the parametric counterparts; the difference concerns only the objective of the inference which focuses on the average in the case of paired and unpaired t test, and looks for a general location shift of the variable’s distribution in the case of the Wilcoxon procedure.

As a sensitivity analysis, we also applied a linear mixed model on log-transformed values of variables but the results were similar and thus only the former have been reported. All tests were two sided and statistical significance was set at p < 0.05. Statistical analysis was performed using R statistical computing software (R Foundation for Statistical Computing, Vienna, Austria).

## Results

From a cohort of 48 prevalent patients on dialysis in a single nephrology unit, 16 patients were excluded because of recent central venous catheter infection (n = 12) and pneumonia (n = 4). Thirty-two patients (mean age was 72 ± 15 years; M = 12) entered the final evaluation. Dialysis vintage was 48 ± 32 months. During the study, vascular access remained patent, no patient was hospitalized or transplanted, and no death was registered. Mean value of Kt/V (measured every two months) was 1.37 ± 0.3 and 1.38 ± 0.5 during the control and study phases, respectively. At the end of the study phase, the level of C-reactive protein (CRP) was lower than at the end of the control phase [0.89 (0.8–1.1) vs. 2.5 (1.0–6.5) mg/l, p < 0.01]. Characteristics of conventional water and water obtained with the ultrafilter are presented in Table 1. Baseline and final routine chemistry and cytokine levels are reported in Tables 2 and 3, respectively. Characteristics of the membrane did not affect the cytokine levels (Table A, supplemental material).

**Table 1:** Characteristics of conventional water and water obtained with ultrafilter

	Water without additional ultrafilter	Water with additional ultrafilter	Reference range
Total microbial counts (CFU/ml)	22	20	100/ml
*Escherichia coli*	0	0	0/100 ml
*Staphylococcus aureus*	0	0	0/100 ml
*Pseudomonas Aeruginosa*	0	0	0/100 ml
Fungi (mycetes)	0	0	0/100 ml
Endotoxin levels (EU/ml) (*Limulus* amebocyte assay; LAL test)	0.125	0	<0.25 EU/ml

Values are the mean of measurements performed every 2 months both during the control phase and study phase

**Table 2:** Biochemistry in control and study phase

	Control phase	Study phase	Treatment effect (95% CI)	p value	Carry-over p value	Period effect p value
Serum creatinine (mg/dl)	8.3 (7.2; 9.5)	8.4 (7; 9.2)	0 (−0.15 to 0.15)	0.895	0.734	0.126
Serum albumin (g/dl)	3.8 (3.6; 4.0)	4.0 (3.8; 4.2)	0.2 (0.08 to 0.31)	<0.001	0.486	1
Total cholesterol (mg/dl)	155 (112.5; 183.3)	171.5 (136.5; 198)	21.32 (−6.5 to 45)	0.132	0.181	1
Triglycerides (mg/dl)	213 (147.8; 276.8)	242.5 (180; 290.5)	21 (19.5 to 25)	<0.001	0.611	0.153
Ferritin (ng/ml)	289.5 (220.8; 333)	275 (205.8; 299.5)	−15 (−48.5 to −4)	0.003	0.865	0.94
Serum phosphorus (mg/dl)	4.3 (3.8; 5.3)	4.4 (3.5; 5.0)	−0.10 (−0.45 to 0.33)	0.678	0.376	0.624
PTH (pg/ml)	311.5 (224.3; 424.3)	349.5 (241.8; 393.8)	17.5 (−73.5 to 116)	0.665	0.97	0.418
Hb (gr/dl)	11.3 (10.9; 11.8)	11.9 (11.8; 12.1)	0.65 (0.50 to 0.90)	<0.001	0.299	0.179

Numbers are median and 95% CI

*CI* confidence interval, *PTH* parathormone, *Hb* hemoglobin

**Table 3:** Cytokine levels in the control and study phase

	Control phase	Study phase	Treatment effect (95% CI)	p value	Carry-over p value	Period effect p value
Pro-inflammatory cytokines						
IL-1 (pg/ml)	9.7 (8.9; 10.6)	9.2 (8.4; 9.8)	−0.78 (−1,12 to −0.5)	<0.001	0.809	0.651
IL-6 (pg/ml)	43.7 (36.7; 50.9)	37.2 (34.7; 44)	−4.36 (−7.09 to −2.57)	<0.001	0.305	0.258
IL-8 (pg/ml)	60.9 (49.4; 74.3)	53.3 (45; 70.5)	−4.35 (−6.1 to −3.04)	<0.001	0.184	0.642
IL-12 (pg/ml)	102.1 (74.3; 124.2)	102.7 (76.3; 125.4)	−0.5 (−1.65 to 0.11)	0.076	0.21	0.91
MCP-1 (pg/ml)	78.8 (73.4; 83.1)	76.3 (71.6; 81.9)	−1.84 (−2.45 to −1.4)	<0.001	1	0.539
TNF-a (pg/ml)	108.8 (98.7; 128.8)	97 (93.8; 113.1)	−7.26 (−11.88 to −4.08)	<0.001	0.752	0.696
SAA (ng/ml)	5949.1 (3248.3; 10,000)	2747.5 (2089.2; 3771.9)	−3491.2 (−4438.5 to−2132.4)	<0.001	0.867	0.445
C5a (ng/ml)	96.2 (78.9; 121.4)	60.3 (48.3; 88.2)	−29.27 (−53.64 to −18.87)	<0.001	0.724	0.402
Anti-inflammatory cytokines						
IL-1ra (pg/ml)	251 (222.2; 295)	255.3 (221.4; 296.4)	5.12 (−0.4 to 10.62)	0.061	0.926	0.616
IL-4 (pg/ml)	14.2 (11; 17.5)	16.2 (13.2; 18.4)	1.35 (0.95 to 1.75)	<0.001	0.224	0.196
IL-10 (pg/ml)	51.4 (45.3; 61.4)	53.3 (50.1; 61.5)	1.22 (−2.9 to 6.69)	0.402	0.254	0.468
IL-13 (pg/ml)	18.5 (15.7; 22.9)	20 (17.6; 25.6)	1.28 (0.69 to 1.89)	<0.001	0.128	0.956
IL-17 (pg/ml)	178.6 (140; 215.1)	183.3 (157.2; 222.8)	3.57 (1.76 to 7.47)	<0.001	0.376	0.491

Numbers are median and 95% CI

*CI* confidence interval, *IL* interleukin, *MCP* monocyte chemoattractant protein, *TNF* tumor necrosis factor, *SAA* serum A-amyloid, *C5a* complement fraction 5

All patients were treated with ESA: 19 with epoietin-alpha (8 with biosimilars, 11 with brand); 10 with darboepoietin alpha; and 3 with methoxy polyethylene glycol-epoetin beta. Changes in Hb concentration, ESA doses and ESA resistance index are reported in Table 4. At the end of the study phase, Hb significantly increased with lower ESA doses and ESA resistance index. The monthly trends in Hb concentration, ESA doses and ESA resistance index are reported in Table B (supplemental material).

**Table 4:** Concentrations of hemoglobin, ESA doses and erythropoietin resistance index (ERI) at Control and Study phase

Variable	Number	Control phase	Study phase	Percent variation study versus control phase
Hemoglobin (g/dl)	32	11.2 ± 0.9	11.9 ± 1.3^●^	+6.25
Epoietin alpha (units per week)	19	11,313 ± 650	8670 ± 554^●^	−23.4
Darboepoietin alpha (units per week)	10	108 ± 111	85 ± 69^+^	−21.3
Methoxy polyethylene glycol-epoetin beta (unit per month)	3	233 ± 130	152 ± 67	−34.8
ERI: EPO (week/BW)/Hb	29	13.1 ± 7.5	9.5 ± 6.0^●^	

Numbers are mean ± SD

^●^ *p* < 0.01

^+^ *p* < 0.05

## Discussion

Current water treatment systems provide high quality dialysis-water. Nonetheless, bacterial products or short bacterial DNA fragments cannot be completely eliminated. Due to their small size, bacterial products or short bacterial DNA fragments easily pass through the dialyzer membrane and into the bloodstream, amplifying the inflammation [6, 9–11]. Several studies have evaluated and quantified the presence of bacterial products or short bacterial DNA fragments both in blood and dialysis-water adopting bacterial DNA primers for sequencing [6–10]; these expensive procedures have established that there is a strict correlation between contaminants and level of inflammation. In contrast, very few interventional studies have been carried out to assess the usefulness of additional devices able to produce ultrapure dialysis-water. In 25 patients on dialysis, ultrafilters reduced the levels of bacterial DNA fragments and endotoxins [11]. However, markers of inflammation were not measured in that study; nonetheless, the increased circulating endotoxin level was associated with systemic inflammation, that was regarded as responsible for vascular stiffness [11]. The latter is frequently observed in different stages of chronic kidney disease and more frequently has been found to be associated to altered mineral metabolism [12–14].

No study has evaluated, to our knowledge, the usefulness of preventive measures such as an additional ultrafilter in minimizing inflammation correlated to dialysis-water by measuring the changes of several circulating markers of inflammation at the same time. The present study evaluated the efficacy of an additional ultrafilter in reducing blood levels of several indicators of inflammation, ameliorating Hb concentration and reducing the level of ESA doses required.

It is significant that blood levels of CRP, SAA, C-5, MCP-1 and of several pro-inflammatory cytokines were lower at the end of the study phase in the present study. The reduction of markers of inflammation was likely promoted by the improved quality of dialysis-water obtained with the additional ultrafilter. Some evidence from the current literature may support this hypothesis despite the fact that levels of bacterial-derived DNA fragments were not assessed in our study. Biological tests adopt cytokine induction to detect the presence of bacterial fragments or bacterial products [9]; plasma endotoxin levels strongly correlate with serum CRP level [11]; high levels of short bacterial-derived-DNA fragments in dialysate increase CRP and interleukin (IL)-6 [6, 10, 15–17]; CPR is produced under the control of various pro-inflammatory cytokines, namely IL-6, IL-1, and TNF-α [1]; circulating bacterial-derived-DNA fragments are associated with higher levels of CRP and IL-6 [6].

Serum levels of SAA, C-5a and MCP-1 have never been taken into account in studies performed to assess the purity of dialysate. SAA is an important acute phase protein with different kinetics from CRP. It has been reported that SAA is a more sensitive indicator of inflammation in some non-cardiovascular inflammatory conditions [18, 19]. It plays a significant role in the atherosclerotic process; in association with other markers of inflammation, SAA contributes to early prediction of cardiovascular mortality [18, 19]. C-5a is a protein fragment released from cleavage of complement component C5; it is a strong inflammatory peptide and a significant pathogenic driver of immuno-inflammatory diseases [20]. MCP-1 is a chemokine that controls the recruitment of leukocytes in inflammation and tissue injury as well as in bone marrow; this suggests that MCP-1 may play a role in hematopoiesis. In addition, MCP-1 may act as factor favoring lipid deposition in the arterial wall as well as being an accelerating factor of inflammation and atherosclerosis [21]. The role of this chemokine in inflammation-mediated diseases is supported by some evidence; in the absence of MCP-1 there was a substantial reduction of lipid deposition in the arterial wall [21]; increased plasma levels of MCP-1 following balloon angioplasty of coronary arteries predicted early restenosis, that may represent an accelerated form of atherosclerosis [21].

Beside the reduction of blood levels of pro-inflammatory cytokines, in our study we observed increased levels of anti-inflammatory cytokines. The production of pro-inflammatory and anti-inflammatory cytokines is controlled by complex feedback mechanisms. Pro-inflammatory cytokines initiate defence against exogenous pathogens. However, overproduction of these mediators can be harmful. As a compensatory mechanism, anti-inflammatory cytokines down-regulate the exacerbated inflammatory process to maintain homoeostasis. The anti-inflammatory cytokines assessed in the present study belong to the class of cytokines activated by an acute or chronic phase of inflammation. For instance, IL-13 induces matrix metalloproteinases that protect against inflammation [22]; IL-1Ra is an acute phase protein important in host defense against endotoxin-induced injury by inhibiting the inflammatory responses caused by IL-1 [22]. We speculate that feedback or counter-regulatory mechanisms were active in our dialysis patients. Therefore, the reduction of inflammatory stimuli by improved dialysis-water triggered a compensatory reaction increasing anti-inflammatory cytokines. Whether the anti-inflammatory response counteracting the pro-inflammatory cytokines may ameliorate outcomes remains to be assessed by other studies.

Interestingly, in the present study marked changes in blood levels of indicators of inflammation were observed while the bacterial count in water-dialysis remained stable after the additional ultrafilter. A plausible hypothesis is that the additional ultrafilter removed bacterial DNA fragments. In keeping with this hypothesis, it is reported that bacterial products or short bacterial DNA fragments have been found in dialysis-water regarded as bacteria-free on the basis of normal levels of endotoxin assessed by the LAL method [6, 7, 23].

It is noteworthy that Hb levels increased at the end of the study phase (by 6%) with lower ESA doses (23% for epoietin alpha; 21% for darboepoietin, and 35% for mircera) and ESA resistance index. We hypothesize that these changes were likely the result of improved inflammation and nutrition.

The trend in conventional indicators of nutrition [24] was not uniform in this study. Indeed, at the end of the study phase there were significant increases in mean blood level of triglycerides and albumin, but not in total cholesterol and serum phosphorus. The latter may explain the nonsignificant increase of mean parathormone (PTH) at the end of the study phase. All together, these data may indicate reduced inflammation as a plausible explanatory factor for the improved nutritional status. A more evident improvement of nutritional status has been reported by others as a positive effect of lower inflammation with ultrapure dialysate [11]. The shorter term of our observation period may explain the inconsistent results of the present study.

### Limitations and strengths

Our study has several limitations. Bacterial fragments/products were not assessed. The study population was not large. The study was monocentric, and the length of observation was not long. Despite these limitations, the study has several strengths such as its cross-over design, and the simultaneous evaluation of pro- and anti-inflammatory cytokines, Hb levels and ESA doses changes.

## Conclusions

The findings of the present study suggest that an additional ultrafilter may reduce activation of proinflammatory cytokines with beneficial effects on anemia. Therefore, further improvement of dialysis-water purity should be pursued to ameliorate the inflammatory status of patients on dialysis. However, these results should be regarded as hypothesis-generating only. Larger studies are mandatory to assess whether the routine use of an ultrafilter is associated to improved survival in dialysis patients.

## Electronic supplementary material

Below is the link to the electronic supplementary material.

Supplementary material 1 (XLSX 12 KB)

Supplementary material 2 (DOC 37 KB)
