# Supplementary material for: Ultrapure dialysis water obtained with additional ultrafilter may reduce inflammation in patients on hemodialysis
Source: J Nephrol. 2017 Aug 23;30(6):795–801. doi: 10.1007/s40620-017-0422-x (PMC5698401; doi:10.1007/s40620-017-0422-x)
Supplement: Supplementary file 2 — Supplementary material 2 (DOC 37 KB) [file 40620_2017_422_MOESM2_ESM.doc]

Table B (supplemental material): monthly trend of Hb and ERI (Epo week dose/bw*Hb)

| **months** | 1 | 2 | 3 | 4 | 5 | 6 |  |
| --- | --- | --- | --- | --- | --- | --- | --- |
| **Control phase** |  |  |  |  |  |  |  |
| Hb | 11.3±1.1 | 11.0±0.9 | 11.1±1.0 | 11.4±0.9 | 11.2±1.3 | 11.2±0.9 |  |
| ERI | 13.3±9.5 | 13.2±8.7 | 13.0±7.9 | 12.9±9.6 | 12.8±10.5 | 13.1±7.5 |  |
|  |  |  |  |  |  |  |  |
| **Study phase** |  |  |  |  |  |  |  |
| Hb | 11.2±1.3 | 11.4±1.5 | 12.1±1.3 | 11.8±1.2 | 11.7±1.2 | 11.9±1.3 |  |
| ERI | 13.4±9.8 | 13.2±8.7 | 9.6±7.1 | 9.3±7.6 | 9.4±6.5 | 9.5±6.0 |  |
|  |  |  |  |  |  |  |  |
| ERI Control  Vs  Stydy phase | NS | NS | 0.001 | 0.001 | 0.001 | 0.001 |  |
